# Supplementary material for: End Sequence Analysis Toolkit (ESAT) expands the extractable information from single-cell RNA-seq data
Source: Genome Res. 2016 Oct;26(10):1397–410. doi: 10.1101/gr.207902.116 (PMC5052061; doi:10.1101/gr.207902.116)
Supplement: Supplemental Material [file supp_gr.207902.116_Supp_Materials.tar.gz › ESAT-esat_v0.1/doc/html/classbroad_1_1pda_1_1seq_1_1clip_1_1_sample_data-members.html]

ESAT: Member List


ESAT


- Main Page
- Related Pages
- Packages
- Classes
- Files

- Class List
- Class Index
- Class Hierarchy
- Class Members

All Classes Namespaces Files Functions Variables Enumerator Pages

- broad
- pda
- seq
- clip
- SampleData

broad.pda.seq.clip.SampleData Member List

This is the complete list of members for broad.pda.seq.clip.SampleData, including all inherited members.

|  |  |  |
| --- | --- | --- |
| data | broad.pda.seq.clip.SampleData | protected |
| expressionByScanPval | broad.pda.seq.clip.SampleData | protected |
| expressionCutoffValue | broad.pda.seq.clip.SampleData | protected |
| firstReadTranscriptionStrand() | broad.pda.seq.clip.SampleData |  |
| fullyContainedReads | broad.pda.seq.clip.SampleData | protected |
| geneAvgCoverage | broad.pda.seq.clip.SampleData | protected |
| genesByChr | broad.pda.seq.clip.SampleData | protected |
| genesByName | broad.pda.seq.clip.SampleData | protected |
| geneScores | broad.pda.seq.clip.SampleData | protected |
| getData() | broad.pda.seq.clip.SampleData |  |
| getEnrichmentOverGene(Gene gene, Annotation window) | broad.pda.seq.clip.SampleData |  |
| getExpressionScanPvalueCutoff() | broad.pda.seq.clip.SampleData |  |
| getFragmentLengthFilterData() | broad.pda.seq.clip.SampleData |  |
| getFragmentLengthGeneCount(Gene gene) | broad.pda.seq.clip.SampleData |  |
| getGeneAverageCoverage(Gene gene) | broad.pda.seq.clip.SampleData |  |
| getGeneCount(Gene gene) | broad.pda.seq.clip.SampleData |  |
| getGeneScanPval(Gene gene) | broad.pda.seq.clip.SampleData |  |
| getLogger() | broad.pda.seq.clip.SampleData |  |
| getOriginalBamFile() | broad.pda.seq.clip.SampleData |  |
| getSampleName() | broad.pda.seq.clip.SampleData |  |
| hashCode() | broad.pda.seq.clip.SampleData |  |
| isExpressed(Gene gene) | broad.pda.seq.clip.SampleData |  |
| logger | broad.pda.seq.clip.SampleData | static |
| maxFragmentLengthData | broad.pda.seq.clip.SampleData | protected |
| maxFragmentLengthGeneScores | broad.pda.seq.clip.SampleData | protected |
| SampleData(String bamFile, boolean firstReadTranscriptionStrand, Map< String, Collection< Gene >> genes, double expressionCutoff, boolean expByScanPval, boolean fullyContained) | broad.pda.seq.clip.SampleData |  |
| sampleName | broad.pda.seq.clip.SampleData | protected |
| setExpressionScanPvalueCutoff(double expressionScanPvalCutoff) | broad.pda.seq.clip.SampleData |  |
| setSampleName(String name) | broad.pda.seq.clip.SampleData |  |


---

Generated on Fri Aug 15 2014 15:11:02 for ESAT by  

 1.8.7
